# Organising Support for Carers of Stroke Survivors (OSCARSS): study protocol for a cluster randomised controlled trial, including health economic analysis

**DOI:** 10.1186/s13063-018-3104-7

**Published:** 2019-01-07

**Authors:** Emma Patchwood, Katy Rothwell, Sarah Rhodes, Evridiki Batistatou, Kate Woodward-Nutt, Yiu-Shing Lau, Gunn Grande, Gail Ewing, Audrey Bowen

**Affiliations:** 1 National Institute for Health Research (NIHR) Collaboration for Leadership in Applied Health Research and Care Greater Manchester (CLAHRC GM), Salford Royal Foundation NHS Trust, Salford, UK; 2 Division of Neuroscience and Experimental Psychology, School of Biological Sciences, University of Manchester, Manchester Academic Helath Sciences Centre (MAHSC), Manchester, UK; 3 Centre for Biostatistics, Institute of Population Health, University of Manchester, Manchester Academic Health Sciences Centre (MAHSC), Manchester, UK; 4 Centre for Health Economics, Division of Population Health, Health Services Research & Primary Care, University of Manchester, Manchester, UK; 5 Division of Nursing, Midwifery & Social Work, School of Health Sciences, University of Manchester, MAHSC, Manchester, UK; 6 Centre for Family Research, University of Cambridge, Cambridge, UK

**Keywords:** Cluster randomised controlled trial, Informal caregivers, Carers, Stroke, Complex intervention, Health service; service user involvement; health economics; qualitative interviews

## Abstract

**Background:**

Stroke often results in chronic disability, with partners and family members taking on the role of informal caregiver. There is considerable uncertainty regarding how best to identify and address carers’ needs. The Carer Support Needs Assessment Tool (CSNAT) is a carer-led approach to individualised assessment and support for caregiving that may be beneficial in palliative care contexts. CSNAT includes an implementation toolkit. Through collaboration, including with service users, we adapted CSNAT for stroke and for use in a UK stroke specialist organisation providing long-term support. The main aims of OSCARSS are to investigate the clinical and cost-effectiveness of CSNAT-Stroke relative to current practice. This paper focuses on the trial protocol, with the embedded process evaluation reported separately.

**Methods:**

Longitudinal, multi-site, pragmatic, cluster randomised controlled trial with a health economic analysis. Clusters are UK services randomised to CSNAT-Stroke intervention or usual care, stratified by size of service. Eligible carer participants are: adults aged > 18 years; able to communicate in English; referred to participating clusters; and seen face-to-face at least once by the provider, for support. The ‘date seen’ for initial support denotes the start of intervention (or control) and carers are referred to the research team after this for study recruitment. Primary outcome is caregiver strain (FACQ - Strain) at three months after ‘date seen’. Secondary outcomes include: caregiver distress; positive caregiving appraisals (both FACQ subscales); Pound Carer Satisfaction with Services; mood (HADs); and health (EQ-5D5L) at three months. All outcomes are followed up at six months. Health economic analyses will use additional data on caregiver health service utilisation and informal care provision.

**Discussion:**

OSCARSS is open to recruitment at the time of article submission. Study findings will allow us to evaluate the clinical and cost-effectiveness of the CSNAT-Stroke intervention, directed at improving outcomes for informal carers of stroke survivors. Trial findings will be interpreted in the context of our embedded process evaluation including qualitative interviews with those who received and provided services as well as data on treatment fidelity. OSCARSS will contribute to knowledge of the unmet needs of informal stroke caregivers and inform future stroke service development.

**Trial registration:**

ISRCTN Registry, ISRCTN58414120. Registered on 26 July 2016.

**Electronic supplementary material:**

The online version of this article (10.1186/s13063-018-3104-7) contains supplementary material, which is available to authorized users.

## Background

Stroke causes a greater range of disabilities than any other chronic condition in the UK [1]. Stroke survivors experience loss of abilities and independence and express concerns about how their condition impacts their partners and family members, who often take on the role of informal caregiver to support personal care and daily living [2, 3]. In the UK alone, informal caregivers for stroke provide care worth up to £2.5 billion per year [4, 5]. This can come at a great personal cost to informal carers, threatening their physical health, connection with family and social networks, finances and emotional wellbeing [6–9].

Identifying and addressing the needs of informal caregivers is a priority at a national level [10–12]. However, several Cochrane reviews highlight considerable uncertainty regarding how best to support stroke caregivers [13–15]. Research suggests that a ‘one-size fits all’ approach to assessment and support is not as beneficial as support that is most closely matched to individuals’ current and specific needs, priorities and preferences [16, 17].

The Carer Support Needs Assessment Tool (CSNAT) intervention [18] is a comprehensive carer-led approach to individualised assessment and support that was developed in the context of palliative care. It includes a staff training package and implementation toolkit. The CSNAT intervention appeared to reduce carer strain in a community palliative care context, when compared to a control of usual care in a before / after stepped wedge design [19]. It also appeared to improve carer psychological and physical health in bereavement in a UK stepped wedge trial [20]. In these pragmatic studies, no changes were made to other support services available for carers between control and intervention periods. Qualitative work with carers [21] and practitioners [22] suggested that CSNAT was highly valued by both groups and made best use of available resources and time when identifying and prioritising needs and supporting carers.

We adapted the original CSNAT intervention and training package for implementation in stroke practice, collectively named CSNAT-Stroke. The adaptation was carried out through close collaboration with carers and a UK stroke service provider organisation. A study-specific Research User Group (RUG) of individuals with experience of caring for a stroke survivor, was set up for OSCARSS and they support study development through regular meetings and representation on the Trial Management Group (TMG). They continue to input to study management while the trial is open to recruitment and thereafter will contribute to interpretation and dissemination of the findings.

In terms of service provider collaborators, a working group of senior Stroke Association staff and their Training and Development department collaborated in development of the staff training and implementation approach used in OSCARSS. The Stroke Association is a stroke specialist provider service with over 200 stroke support services throughout the UK. Services are organised flexibly to meet requirements of the local population; practice therefore varies across different services according to availability and preferences. Many services are embedded in hospitals and referrals for support are primarily received from the National Health Service (NHS) soon after the stroke event; although individuals can be referred – or self-refer – at any time after stroke. All OSCARSS research sites/clusters are drawn from Stroke Association services.

### Trial aim and research questions

The primary aim of OSCARSS is to determine the effectiveness of the CSNAT-Stroke intervention for carers of stroke survivors, when compared to a usual care control. The primary research question is: does the intervention reduce caregiver strain (as measured by the strain subscale of the Family Appraisal of Caregiving Questionnaire (FACQ) [23]), when compared to control?

Secondary research questions address whether the intervention:

- reduces perceived caregiver distress (subscale of FACQ) [23];
- improves: carer perceptions of their health (EQ-5D-5 L) [24] and wellbeing (Hospital Anxiety and Depression Scale (HADS) [25]; positive caregiving appraisals (subscale of FACQ) [23]; and satisfaction with services (Pound Scale) [26];
- leads to less economic burden for carers and society (as measured by an adapted version of the Service Receipt Inventory [27] that records use of health services and informal care provision).

## Methods

OSCARSS is a longitudinal, pragmatic multi-site cluster randomised controlled trial (cRCT) with a health economic analysis and nested process evaluation. Cluster randomisation is essential to avoid contamination as we are evaluating delivery of an intervention within a service, sometimes by a team.

**Fig. 1:**
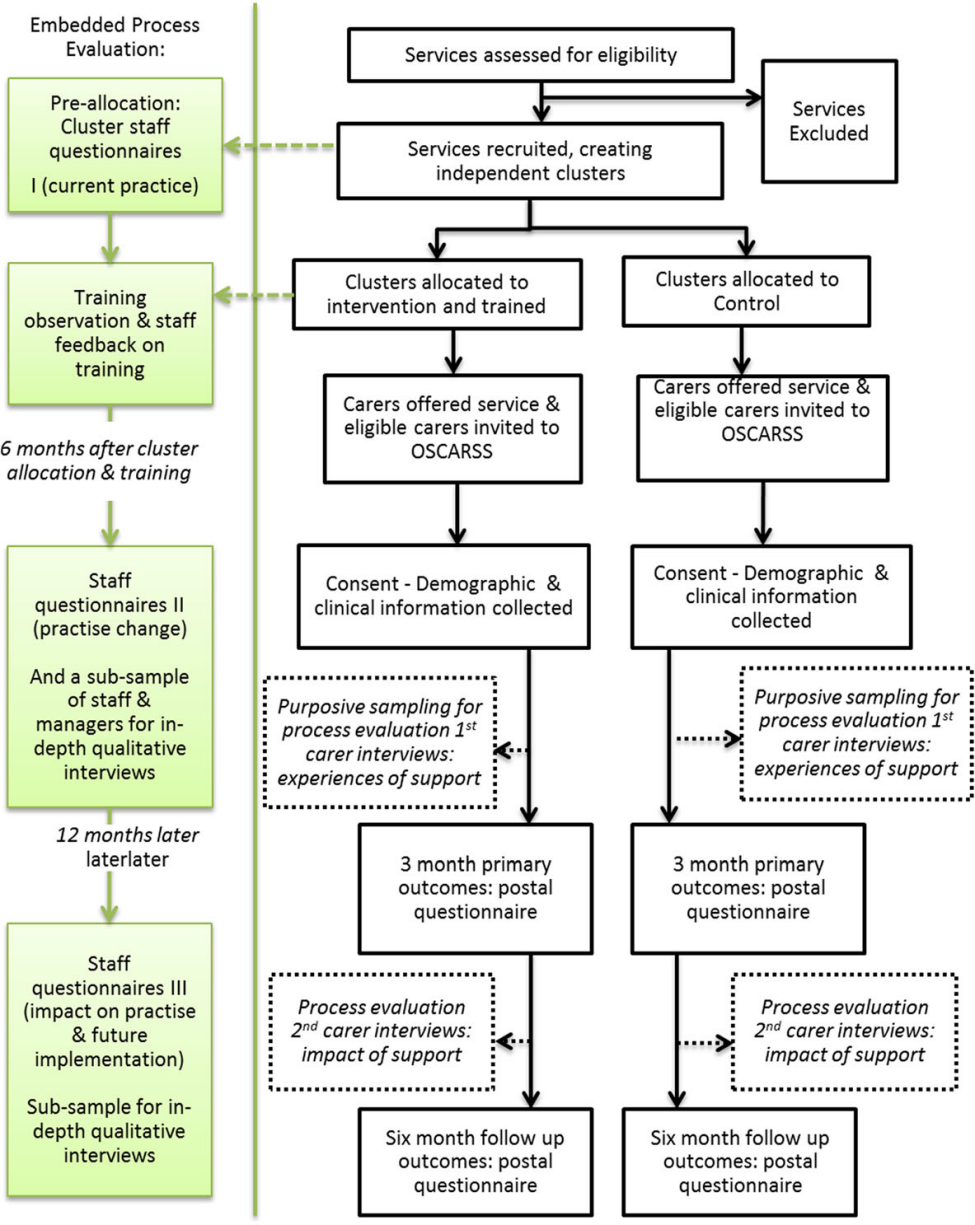
Outline of the OSCARSS study process. For contextual information, this figure includes cRCT processes (*middle*) as well as components of the embedded process evaluation: carer interviews (*dotted line boxes* within middle figure); staff and manager surveys and interviews (*left*)

Not described in detail in this protocol is an embedded process evaluation that includes survey data, service delivery records and qualitative data. In brief, data collected from service providers (staff and managers) will explore intervention implementation and workforce behaviour change. Interviews with service recipients (carer research participants) will explore their experiences of support (intervention or control) and the types of support inputs identified and prioritised by them. Figure 1 shows these parallel components of OSCARSS but the cRCT and health economics are the focus of this paper. The process evaluation, which will be invaluable in providing the context for interpretation of the trial findings, will be described elsewhere.

A Standard Protocol Items: Recommendations for Interventional Trials (SPIRIT) checklist is provided as Additional file 1.

### Trial clusters: inclusion criteria and randomisation

Eligible clusters are defined as UK stroke specialist provider services that:

- include face-to-face contact with carers (excluded are services that only provide telephone support);
- have capacity to engage in OSCARSS (excluded are services participating in any other stroke carer-related research);
- are likely to have at least five new client referrals per month. Clients include both stroke survivors (who are likely to have associated carers) and carers directly. This ensures that a new system of working can be operationalised and well-established and that research resources required for training and monitoring sites are justified;
- are independent of other clusters. If staff across services shared client caseloads, they would be aggregated to form one cluster to avoid the risk of between-group contamination. Conversely, individual staff within services could form independent clusters if they work independently, without sharing caseloads.

Clusters are recruited by the CLAHRC GM research team before randomisation, to ensure allocation concealment. Clusters are block randomised to intervention or control at the ‘site’ level with dichotomised stratification for ‘size of service’ (high / low -based on historic data about client caseloads) using random blocks. Neither the clusters nor the researchers know the block sizes when recruiting sites. The trial statistician is provided with an anonymised list of recruited clusters and randomises them using STATA programme, including the ‘ralloc’ add-on.

The research team is blinded to allocation as far as possible, but front-line team members could become unblinded when observing staff training (delivered after randomisation) or when supporting sites to engage in the study.

Carer research participants are blind to allocation; they receive support by the provider organisation in both arms of the trial, but the nature of support is different according to allocation to research intervention or control. Carers are not consenting to randomisation but to follow-up.

### Intervention

The CSNAT-Stroke is the research intervention, described briefly here. All intervention materials, including training handbook and instructional videos, will be made available after the trial when treatment fidelity and adherence will be reported.

**Fig. 2:**
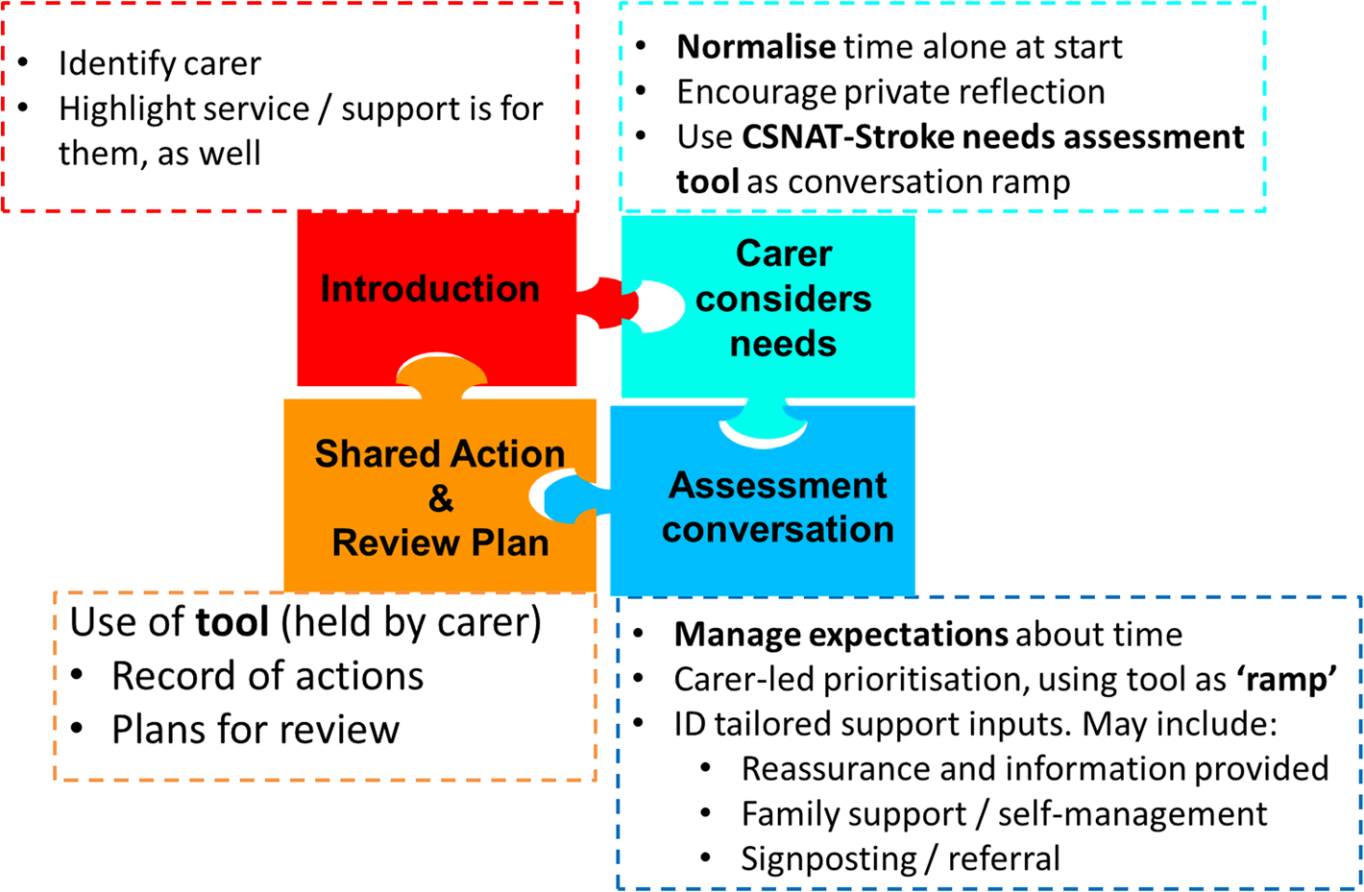
Basic visual representation of the CSNAT-Stroke intervention

CSNAT-Stroke provides a structured, standardised approach to offering an evidence-based needs assessment for carers, which is distinct from the stroke survivor. It involves the use of a single-page assessment tool organised into broad domains of need and a written action plan for review. CSNAT-Stroke is predicated on staff behaviour change and follows a general process that can be flexibly applied whenever the staff member has contact with a carer. It is facilitated by a staff training and implementation package. CSNAT-Stroke promotes a carer-led, practitioner-facilitated approach to identifying and implementing support inputs that are directly derived from the needs assessment. Table 1 summarises the intervention process with Fig. 2 as a basic visual representation of the intervention. As described in our dissemination plan, we will report using the Template for Intervention Description and Replication (TIDieR) guidelines [28].

**Table 1:** Summary of CSNAT-Stroke intervention

Step	Description	Timing and duration	Mode
Introduction	Carers identified and assured that support is available	Point of referral to service; ≈ 5 min duration	Telephone or face-to-face (inpatient settings or home), depending on referral
Carers consider needs	CSNAT-Stroke needs assessment tool introduced. Carers encouraged to take independent time to consider and complete, indicating domains in which they need more support	At the point of contact; ≈ 5 min duration	Face-to-face (may be sent by post for follow-ups)
Assessment conversation	Using the CSNAT-Stroke tool as a ‘conversation ramp’, carers supported to prioritise the domains most important to them currently: to identify their individual needs within those domains and the type of supportive input they would find helpful. Support may be directly delivered by practitioner at this time (e.g. reassurance and information) but family support, signposting or referral to other agencies may also be included	During support contact. Duration dictated by time available; ≈ minimum 10 min, with ‘set up’ regarding time available to manage expectations	Typically face-to-face but possible by phone for follow-up contact
Shared action and review plan	Actions to address needs are recorded on a paper tool and, if appropriate, a plan is agreed regarding review / update on actions carried out	Following assessment conversation	Carers given hard copy action plan. Staff records in service database

### Carer research participants: inclusion criteria and recruitment processes

Adult (aged > 18 years) informal carers of stroke survivors are eligible if they:

- are referred to participating clusters;
- receive at least one face-to-face support contact (regardless of the resultant level of support / need, e.g. support may be a single face-to-face visit, with follow-up support by telephone);
- are able to communicate in English (facilitated by supportive communication techniques); and
- are ‘active’ in their caring role at the time of study entry, i.e. the stroke survivor being cared for is alive.

Following the first face-to-face contact, i.e. after support has been delivered according to intervention or control, staff provide a brief OSCARSS information leaflet and ask carers if they would like to be referred to the research team to find out more about potential study participation. Carers are told that the service is being evaluated, but they are not told about the randomised trial (blinding). The opportunity for study referral is offered, even if a carer does not go on to receive further support from the service. Carers can be given up to six weeks to decide about study referral.

If carers accept referral, their details are securely passed to the research team who make first contact by phone, introducing the study and providing full study information by post to seek consent. This process ensures a clear separation between the research and the provision of support (either research intervention or control).

Informed consent is sought by researchers trained in Good Clinical Practice (GCP) and using approved documents for information and consent, which were co-designed with carers via the OSCARSS RUG. Participant information and consent materials are available on request from the authors and will be published at trial end. The right to refuse participation without giving reasons is respected and research participants remain free to withdraw at any time from the study without giving reasons and without prejudicing further support.

### Data collection

**Fig. 3:**
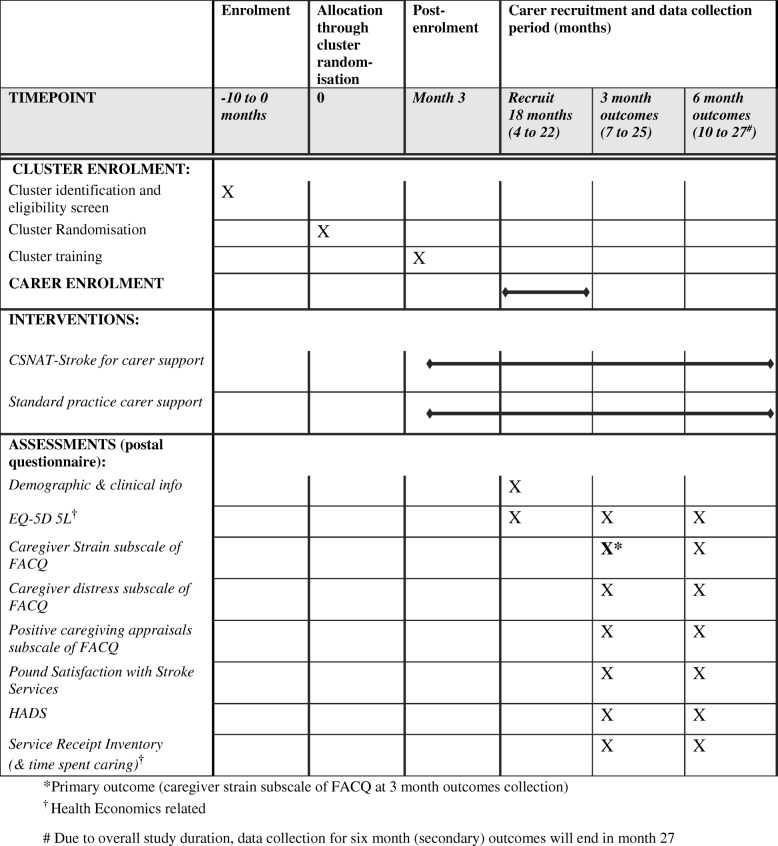
SPIRIT figure

The schedule of data collection and the outcome measures to be collected are shown in Fig. 3, the SPIRIT figure, with more detail below.

### Carer self-report measures

Demographic and clinical characteristics, related to the carers and their cared-for stroke survivors, are collected at study entry, along with EQ-5D-5 L [24]. These data are not strictly speaking baseline, as support (either intervention or control) has already been initiated at the point of study referral. Staff provide a ‘date first seen’ when referring carers to the study and this is considered the ‘start date’ for intervention or control.

Initial and follow-up outcomes are sought three and six months after ‘start date’, respectively. The time difference between study entry and outcomes collection will not necessarily be exactly three and six months, since the recruitment process takes some time after referral (and, as above, referrals can be received up to six weeks from ‘start date’). In cases where carers request more time to make a decision about consent, study entry data and three-month outcomes can be collected simultaneously. To allow sufficient time for reminders and return post, initial outcomes can be returned any time up to 4.5 months from ‘start date’. Follow-up outcome data are sought at six months and can be returned any time up to 7.5 months from ‘start date’. Due to the study end date, we can only collect six-month (secondary) outcomes up to month 27. A study database auto-generates all prompts for data collection and, to improve retention, phone calls engage participants before any postal packs are sent. Thank you notes also advise when participation is complete and that a final report on results will be sent at study close (see ‘Dissemination plan’).

All measures are described below and are completed by carers via self-report postal questionnaire. Carers are given the option to complete over the phone with telephone support from a researcher:

- FACQ [23]: the caregiver strain (primary outcome at the three-month collection point) and caregiver distress subscales assess the negative impact of caring, while the positive appraisals subscale assess the positive impact of caring. The strain subscale of FACQ was used to successfully evaluate the effectiveness of the original CSNAT in a palliative care context [19] and is chosen as the primary clinical endpoint as it directly addresses the primary research aims. In addition, the OSCARSS RUG agreed that caregiver strain was most likely to be alleviated through this intervention and felt that, when compared to other candidate caregiver strain or burden scales, FACQ was more relatable and more likely to be completed through postal questionnaire;
- caregiver’s perceived quality of support and satisfaction with services will be assessed using the Pound Carer Satisfaction with Stroke Services Scale [26];
- carer wellbeing and health will be assessed using the HADS [25] and EQ-5D-5 L [24], respectively;
- for health economic analysis, an adapted version of the Service Receipt Inventory [27] will record use of health, social care and third sector services, as well as the amount and nature of informal care provision.

### Service delivery records

Staff with access to the clusters’ in-house data management systems will securely provide study-specific data to the research team for consented carers. This will include: the dates, types and durations of support contacts delivered; and standardised entries from staff pertaining to needs identified and actions taken during support contacts. Support contacts include both direct and non-direct contact (e.g. liaison with external agents). Health economics analysis will include ‘service delivery costs’ for each consented carer, based on these data, by valuing support time using service provider full costs. As well as data specific to consented carers, the research team will be securely provided with fully anonymised service delivery records for all clients in participating services / clusters (intervention and control). These records will contain no personal client data but will include: the number, duration and type of contacts completed by coordinators; and categories of needs identified and actions completed. These data will support an economic understanding of whole service delivery across participating clusters (comparing intervention to control) and an exploration of how representative OSCARSS participants are of all cluster clients.

#### Sample size

The primary outcome is the Caregiver Strain subscale of the FACQ [23] at three months after intervention / control (see also Fig. 3). This subscale scale consists of eight questions, each worth a maximum of 5 points, and can be reported as a mean score per question (maximum score = 5.0) or total number of points (maximum score = 40 points). Cooper et al. [23] reported a mean (SD) of 3.13 (0.87) on this subscale on a study of 160 participants. In their trial to assess the impact of the CSNAT intervention in the palliative care setting, Aoun [19] reported a standardised effect size on the FACQ caregiver strain subscale of 0.348 which corresponds to a difference of 0.31 on the mean score. Based on empirical data from similar settings, we do not expect the intraclass correlation coefficient (ICC) to be > 0.05 (https://www.abdn.ac.uk/hsru/what-we-do/tools). In fact, TRACS, a cluster randomised trial which trained carers to provide care to stroke survivors [27], reported ICCs of 0.013 for caregiver burden.

Table 2 shows the sample sizes to achieve 80% power, assuming at least 16 active clusters per arm and SD = 0.9.

**Table 2:** Sample size projections

ICC	Effect size = 0.31 unit change in mean score (2.5 points change on total score)
0	288
0.01	320
0.025	384
0.05	512
0.075	800

Our minimum target is 320 carers providing primary outcomes at three months. This would allow us 80% power to detect effect sizes of 0.31 or more for ICCs ≤ 0.01, and effect sizes of ≥ 0.375 for ICCs of ≤ 0.05. We assume a retention rate of 80% between consent and primary outcomes, which means we require a minimum of 400 consented carers.

An optimum sample size of 512 (640 consented carers) would allow us 80% power to detect effect sizes of ≥ 0.31 for ICCs ≤ 0.05 and would allow us to detect effect sizes of ≤ 0.25 for an ICC of 0.01. We would cease recruitment if we hit this figure before the planned recruitment end date.

Sample size calculations were carried out using the *clsampsi* function in STATA.

### Statistical analysis

#### Adverse events

This study’s intervention is low risk, primarily involving staff behaviour change when supporting carers within their role. Serious adverse events (SAEs) are an inherent part of an active caregiving role (e.g. musculoskeletal injury; new medical problems or deterioration of existing medical problems, including depression). It is possible that these could lead to hospitalisation, prolongation of existing hospitalisation, disability / incapacity or death. As such, they are expected SAEs; there are no SAEs that we predict will be related to the research intervention. All adverse events (AEs) will be recorded. SAEs will be reported to the Research Ethics Committee (REC) within 15 days if the Chief Investigator believes they might be related to the research and unexpected.

#### Analysis, including economic evaluation

A full and detailed statistical analysis plan (SAP), including information on how any missing data will be managed, is included as an Additional file 2.

Analysis of the primary outcome comparing intervention and control at three months will be carried out on the basis of intervention to treat (ITT) and performed using a multilevel regression model, with a random intercept for ‘site’ to take into account clustering and a fixed covariate for ‘intervention’ along with adjustment using the following fixed individual level covariates: stroke severity of cared-for person (as rated by carer), time post-stroke, age of carer, health of carer at study entry (as indicated by self-reported pre-existing long-term health conditions) and the following cluster level covariates; size of service, pre-existing knowledge/experience of staff delivering support. By the design of this cluster randomised trial, recruitment of individual carers takes place after randomisation and therefore we are at risk of selection bias. We plan to adjust for baseline covariates in an attempt to control for any baseline imbalance. Similar analysis will be used for all numeric secondary outcome measures.

The mean number of carers per cluster, the mean number of support contacts per carer per cluster and the mean duration of contacts per carer per cluster will be compared between control and intervention groups using t-tests. We would not expect these variables to have appropriate distributions for analysis using a linear mixed model.

Sensitivity analyses will explore any potential bias in the analysis of the primary outcome measure and examine how robust the findings are:

i. without adjustment for covariates;
ii. per protocol;
iii. combining three-month and six-month month data: using ‘time’ and ‘time by group interaction’ as fixed covariates, all available three-month and six-month data will be combined for the Caregiver Strain subscale of the FACQ. This will allow us to explore how caregiver strain changes over time and whether any effect of the intervention changes over time;
iv. multiple imputation: using multiple imputation to replace missing values on the primary outcome measure using the following covariates: stroke severity of cared-for’s stroke; time after stroke; age of carer; pre-stroke health of carer (as per Royston [29]);
v. excluding delayed responses: excluding any data from individuals who return their three-month outcome data later than 4.5 months after ‘date seen’ or six-month outcome data later than 7.5 months after ‘date seen’;
vi. removing carer dyads: where multiple carers of the same stroke survivor have provided outcome data; excluding data from the second and subsequent carers linked to the same stroke survivor.

Data relevant to the Health Economics analysis will include the Service Receipt Inventory, number of support contacts delivered per carer, informal care provision estimates and EQ-5D-5 L. Trial health economists will attach costs to questionnaire items and support contacts to allow a comparison across research intervention and control arms of the trial. Prognostically important variables such as carer health and demographics will be factored into an analysis comparing use of healthcare services, with severity of stroke survivor factored in to an analysis comparing time spent caring.

### Data management and monitoring

All information collected is kept strictly confidential. Information will be held securely on paper in locked filing cabinets and electronically on encrypted servers. All data are anonymised as early as possible, with carers assigned a unique identifier as soon as they are entered into the database. If a participant withdraws consent at any time, their research data will remain on file and will be included in the final study analysis, unless otherwise requested. If a withdrawing participant agrees to receive a final report summarising the results of the study, their contact information will be held on file for these purposes and will be deleted once the final report is sent.

Standard Operating Procedures (SOP) for data entry processes ensure consensus in interpreting ambiguous data. The SOP also outlines data checking for quality and is available on request. Delegation logs determine which study staff are trained and assured to carry out specific tasks, including data entry.

The Research Team will form a Trial Management Group (TMG) and a Trial Steering Committee (TSC). The TSC will be chaired by and include independent members as well as key trial personnel. Data to be regularly monitored will include: individual level study-entry demographic and clinical variables; and cluster level data related to referrals and recruitment. The TMG and TSC will consider recruitment and balance across the intervention and control arms throughout the study. After four months of carer recruitment, the TSC met to consider these data to make a recommendation as to whether the trial should be allowed to continue, continue with modification or be discontinued (they decided on the former). A TSC charter outlining roles and responsibilities is available on request.

## Discussion

This paper describes the protocol for a novel trial exploring clinical and cost-effectiveness of a pragmatic intervention to support and empower informal carers of stroke survivors. It differs from TRACS [27] in that OSCARSS trains staff to support carers’ own needs whereas the focus of TRACS was to train carers to perform the caring role. The OSCARSS intervention has been adapted from an existing approach used successfully in palliative care settings.

All aspects of the study have been designed in collaboration with key stakeholders, including carers themselves who form our study specific RUG, and stroke professionals who deliver the support. We believe this collaboration strengthens the study, including optimising recruitment processes and outcome measurement.

There are also some challenges to address, including the lack of baseline measures to explore change in outcomes, which the randomised design helps overcome. In terms of outcomes, the majority of our clinical endpoint data will be based on carer self-report, using measures that have been carefully selected through consultation with literature and co-development with service users. The intervention aims to provide individualised carer support and reduce the negative impact of caregiving, but our carer eligibility criteria are extremely inclusive and do not require diagnosis of depression, anxiety or similar. As such, hard clinical endpoints requiring professional assessment would be inappropriate in this pragmatic trial.

The decision to widen the time window for returning the postal questionnaire is a pragmatic one but may increase variability in when we measure outcomes. This will be adjusted for, as needed, with sensitivity analysis. Cluster randomisation is essential to avoid contamination as we are evaluating delivery of an intervention within a service but leads to potential for differential recruitment as allocation is known in advance of consent. Methods to overcome this have been outlined above and in the SAP but in addition, all cluster staff are given similar training with regards to recruitment and record keeping and are regularly engaged with by the research team and service managers to encourage consistent referrals to the study. Generalisability will be explored through comparing characteristics of our sample to anonymised data related to national caseloads of the service provider.

This paper has focused on the cRCT and health economics, but it is strengthened by an embedded mixed-methods process evaluation to ensure a contextualised interpretation of our findings. The process evaluation will be described fully elsewhere but includes: implementation of the research into practice; sustainability of the research intervention; and the effect of research team on staff behaviour. The research intervention requires staff behaviour change and the pragmatic design leads to anticipated challenges exploring intervention fidelity, which the process evaluation will also help overcome. Semi-structured qualitative interviews will explore staff and carer experiences of delivering and receiving support, respectively. Interviews are completed with purposively sampled participants, considering demographic variables, arm allocation and geographical location. Interviews and focus groups will also be completed with service provider managers and senior leadership teams. The process evaluation is overseen by expert implementation and qualitative researchers who were not involved in the trial design.

Overall, OSCARSS will provide pragmatic data on future healthcare development for supporting carers of stroke survivors. Health economics components will allow exploration of costs with a view to providing a costed service specification to directly inform service improvements. The model for adapting and implementing the research intervention through collaboration could be applied to other health conditions and settings.

### Dissemination plan

The findings from OSCARSS will be published in scientific journals using the following guidelines: Consolidated Standards of Reporting Trials (CONSORT) guidelines for cRCTs [30]; Template for Intervention Description and Replication (TIDieR) guidelines for intervention description [28]; and Consolidated criteria for reporting qualitative research (COREQ) guidance for qualitative research [31]. Trial findings will also be written up in accessible, lay-friendly language and disseminated to research participants and on the NIHR CLAHRC Greater Manchester website. A study-specific event to disseminate to all stakeholders will be held and we will disseminate to wider audiences through local, national and international conferences. Implementation activities will be finalised once the results are known.

### Trial status

Clusters were randomised in September 2016 and trained in January 2017, when carer participant recruitment began. The first carer was enrolled on 17 January 2017. Recruitment is ongoing (at the time of journal submission) and will be completed by 31 July 2018.

## Additional files

Additional file 1: SPIRIT 2013 Checklist. (DOC 141 kb)

Additional file 2: Statistical Analysis Plan. (DOCX 59 kb)

## Abbreviations

AEs: Adverse events
CLAHRC: Collaboration for Leadership in Applied Health Research and Care
COREQ: Consolidated criteria for reporting qualitative research
cRCT: Cluster randomised controlled trial
CSNAT: Carer Support Needs Assessment Tool
FACQ: Family Appraisal of Caregiving Questionnaire
GCP: Good Clinical Practice
HADS: Hospital Anxiety and Depression Scale
ICC: Intraclass correlation coefficient
ISRCTN: International Standard Randomised Controlled Trial Number
MAHSC: Manchester Academic Health Sciences Centre
NHS: National Health Service
NIHR: National Institute for Health Research
OSCARSS: Organising Support for Carers of Stroke Survivors
REC: Research Ethics Committee
RUG: Research User Group
SAE: Serious adverse event
SAP: Statistical analysis plan
SD: Standard deviation
SOP: Standard Operating Procedure
SPIRIT: Standard Protocol Items: Recommendations for Interventional Trials
TIDIER: Template for Intervention Description and Replication
TMG: Trial Management Group
TSC: Trial Steering Committee
UK: United Kingdom
