# Supplementary material for: Organising Support for Carers of Stroke Survivors (OSCARSS): study protocol for a cluster randomised controlled trial, including health economic analysis
Source: Trials. 2019 Jan 7;20:19. doi: 10.1186/s13063-018-3104-7 (PMC6323775; doi:10.1186/s13063-018-3104-7)
Supplement: Supplementary file 2 — Statistical Analysis Plan. (DOCX 59 kb) [file 13063_2018_3104_MOESM2_ESM.docx]

**OSCARSS Statistical Analysis Plan**

**Contents**

| **Section** | **Title** | **Page** |
| --- | --- | --- |
| 1 | **Trial Summary**  1.1 Outcomes (cRCT)  1.2 Analysis population | 2 |
| 2 | **Study Population**  2.1 Recruitment and retention  2.2 Study entry characteristics  2.3 Differential Recruitment | 3 |
| 3 | **Statistical Principles**  3.1 Randomisation  3.2 Sample Size  3.3 Confidence and significant levels  3.4 Adherence and protocol violations  3.5 Interim Analyses  3.6 Timing of Analyses  3.7 Missing Data  3.8 Assumption Checking  3.9 Data Validation | 4 |
| 4 | **Analysis of Outcomes**  4.1 Data manipulations  4.2 Data analysis  4.3 Sensitivity analyses  4.4 Sub-group analysis  4.5 Safety data  4.6 Statistical Software | 7 |
| 5 | **Health Economics**  5.1 Outcomes  5.2 Missing data  5.3 Analysis of Outcomes  *5.3.1 Unit Costs*  *5.3.2 Data Manipulations*  *5.3.3 Data Analysis*  *5.3.4 Sensitivity Analysis of Economic Evaluation*  5.4 Secondary analyses | 9 |
| 6 | **References** | 12 |

1. **Trial summary**

The main research aim is to determine the effectiveness of the CSNAT-Stroke intervention for carers of stroke survivors, when compared to a control of standard practice. OSCARSS uses a mixed methodology, longitudinal, multi-site cluster randomised controlled trial (cRCT) with a health economic analysis and embedded process evaluation collecting quantitative and qualitative data.

This document refers to statistical analysis within the cRCT (main components of sections 1 to 4) and Health Economics (section 5). The SAP should be read in conjunction with the protocol and is to be applied to a clean dataset.

**Design:** Cluster randomised trial randomised at the ‘site’ level with stratification for ‘size of service’.

**Population:** Adult carers of stroke survivors referred to participating Stroke Association services

**Intervention:** The CSNAT-Stroke intervention which provides a structured, standardised approach to carer support, delivered by Stroke Association.

**Control:** Current level and mode of carer support from Stroke Association

- 1. Outcomes:

**Primary outcome measure:**

Caregiver Strain subscale of the FACQ at 3 months post study intervention / control start date

**Secondary outcomes:**

Caregiver Strain subscale of the FACQ at 6 months post study intervention / control start date

All the following at 3 & 6 months post-intervention / control start date

- Caregiver distress subscale of FACQ
- Positive caregiving appraisals subscale of FACQ
- Pound Satisfaction with Stroke Services Scale
- Hospital Anxiety and Depression Scale

(see Health Economics section 6 for additional)

- 1. Analysis population:

The main analyses of the primary and secondary outcomes will be carried out on the basis of intention to treat (ITT). The ITT population consists of all recruited participants who have an outcome measurement (see Section 3.7 for handling missing data). The analysis population may occasionally include multiple carers linked to the same stroke survivor. The analysis population may occasionally include carers who have returned their 3 month and 6 months outcome data outside the expected windows of 3 to 4.5 months and 6 to 7.5 months respectively.

Participants will be analysed according to the group to which their cluster was randomised, independent of fidelity to the intervention or control at a cluster or individual level.

1. Study population
   1. Recruitment and retention

A CONSORT diagram will summarise cluster recruitment and recruitment of carers within clusters, including screening and retention. Reasons for withdrawal and loss to follow-up will be tabulated by treatment arm.

- 1. Study entry characteristics

The variables listed below will be described, both overall and separately for the two randomised groups. No statistical inference will be applied to these comparisons.

**Cluster (site) level**

- Size of service, as indicated by number of stroke survivors referred during a fixed 9 month historical period (median, range)
- Amount of staff training / experience, as indicated by several variables including number of years of employment within Stroke Association and amount of training / qualifications (median, range)

**Individual carer level**

- Age (median, range, IQR)
- Sex (n,%)
- Ethnicity (n,%)
- Employment status (n,%)
- Marital status (n,%)
- Living status (alone; with stroke survivor; or with family/significant others) (n,%)
- Time since stroke of stroke survivor (median, range, IQR)
- Relationship to Stroke Survivor (n,%)
- Carer-perceived ‘independence’ of the SS (indicator of stroke severity from PoV of carers) (median, range, IQR)
- Highest level of formal education completed (n, %)
- Long-term health conditions (n, %)
- Any caring activities pre-stroke (n, %)

3.3 Differential Recruitment

By necessity, the recruitment of carers to the OSCARSS study occurs after randomisation and there is a risk of differences between control and intervention arms in terms of (a) the rate of recruitment and (b) the type of carers recruited, as observed by Farrin et al (2005). The following methods have been employed in an attempt to minimise the risk of bias due to differential recruitment.

1. Participants are not made aware of the randomised element of the study or existence of ‘intervention’ and ‘control’ arms
2. Stroke association staff in both arms are given similar training with regards to recruitment and record keeping. Staff are educated in the importance of approaching all eligible carers with equal encouragement.
3. The primary analysis will be adjusted for baseline variables, as recommended by Giraudeau(2009).
4. The rate of recruitment and baseline characteristics across arms will be monitored carefully for the duration for the trial. These data will be presented to the Trial Steering committee, and in the event of serious differential recruitment their independent advice will be taken about data analysis. Options would include (a) using propensity score methods (Leyrat 2013) (b) not analysing outcome data.
5. **Statistical principles**
   1. Randomisation

Stroke Association sites are assigned to either the intervention or control group with a 1:1 allocation. Sites will be divided into 4 equal strata based on size of clusters, measured using historical data on the ‘number of stroke survivors referred per month’ over a nine month period. Each stratum will be allocated to intervention or control groups using random blocks of size two. For randomisation and stratification, the trial statistician will use STATA programme, including the ‘ralloc’ add-on to produce randomisation lists. All clusters will be recruited before randomisation is performed. The initial randomisation list will produce allocations for 36 clusters.

To replace clusters that drop out or fail to recruit, new clusters may be allowed to join OSCARSS after the initial start date. To accommodate new clusters recruited after the start of OSCARSS, a second randomisation list will be produced to allocate up to 16 clusters using the same procedures as above. New clusters will be allocated to strata as they are recruited using the same range of values of ‘number of stroke survivors per month’ for strata as were observed in the initial randomisation schedule.

- 1. **Sample Size**

The primary outcome is the Caregiver Strain subscale of the FACQ, collected at 3 months after carers are first seen by Stroke Association staff (intervention / control). This subscale scale consists of 8 questions, each worth a maximum of 5 points, and can be reported as a mean score per question (maximum score = 5.0) , or total number of points (maximum score =40 points). Cooper et al (2006) reported a mean (SD) of 3.13 (0.87) on this subscale on a study of 160 participants. A reduction of 0.375 units on this 8 item subscale would correspond to improvement of 1 point on each of 3 items. A reduction of 0.25 units would correspond to an improvement of 1 point on each of 2 items. In their trial to assess the impact of the CSNAT approach in the palliative care setting Aoun et al reported a reduction of 0.31 units (2.5 points different on the total score) on the FACQ caregiver strain subscale.

## Table 1: sample size projections

Based on N of primary outcomes collected to achieve 80% power, assuming 16 equal clusters per arm and SD=0.9

| **ICC** | **Effect size (units change on mean score of primary outcome)** | | |
| --- | --- | --- | --- |
|  | **0.375**  **(3 points)** | **0.31**  **(2.5 points) Aoun et al.** | **0.25**  **(2 points)** |
| **0** | **224** | **288** | **448** |
| **0.01** | **224** | **320** | **512** |
| **0.025** | **224** | **384** | **672** |
| **0.05** | **288** | **512** | **1312** |
| **0.075** | **352** | **800** |  |

Based on empirical data from similar settings we do not expect the ICC to be greater than 0.05 (<https://www.abdn.ac.uk/hsru/what-we-do/tools>). In the TRACS cluster randomised trial of a training needs programme for stroke carers Forster et al (2013) reported ICCs of 0.013 for their caregiver burden scale.

We assume a retention rate of 80% between consent and primary outcomes (based on figures to date).

Our minimum target is therefore 320 carers providing primary outcomes. This would allow us 80% power to detect effect sizes of 0.31 or more for ICCs of 0.01 or less and effect sizes of 0.375 or more for ICCs of 0.05 or less. With retention rates of 80%, this would require 400 consented carers.

An optimum sample size of 512 (640 consented carers) would allow us 80% power to detect effect sizes of 0.31 or more for ICCs of 0.05 or less, and would allow us to detect effect sizes of 0.25 or less for an ICC of 0.01. We would cease recruitment if we hit this figure before the planned finish date.

- 1. Confidence and significance levels

Analyses will be conducted using two-sided 95% confidence intervals and the 5% significance level. All analyses will be adjusted for clustering by site. No formal adjustments will be made for multiplicity as there is a single primary analysis.

- 1. Adherence and protocol violations

1. Adherence.

Adherence to the intervention will be assessed descriptively by a number of different methods looking at both a cluster and individual level using: the client management database, observation of staff training sessions, staff questionnaires and qualitative interviews.

1. Protocol deviation and violation

Possible protocol deviations include:

- Recruited sites who fail to attend planned training activities
- Recruited sites who fail to follow protocol for intervention delivery
- Recruited sites who fail to follow protocol for participant recruitment or data collection
- Recruited carers who return outcomes data outside of intended date windows
- Recruited carers who become unblinded
- Individuals who return their 3 months outcome data later than 4.5 months post date seen or 6 month outcome data later than 7.5 months post date seen.

Major protocol violations will be presented to the Trial Steering Committee (TSC) as soon as they are brought to the attention of the trial team. Recruitment data, study entry characteristics and data from the Process Evaluation (described in the protocol) will be reviewed by TSC before any outcomes are analysed to determine whether any clusters (or staff members within a cluster) should be categorised as deviating / non-adhering to the extent that they are likely to confound results.

All protocol deviations and decisions about adherence will be carefully documented, as will any attempts to work with sites to address these issues. No formal statistical testing will be undertaken; decisions made here will influence the ‘per protocol’ analysis (see Sensitivity Analyses section 4.3)

- 1. Interim analyses

Interim analyses will be undertaken of baseline data, recruitment and retention. No interim analyses of outcome data will be conducted and no adjustment will be made for possibility of early stopping due to data monitoring considerations, unless specifically asked for by the independent TSC or ethics committee.

- 1. Timing of analyses

All carer-reported data collected by postal questionnaire and relevant to the cRCT and analyses described in this SAP are scheduled to be collected as shown in Table 2. No outcome analyses will be undertaken until 5.5 months after recruitment to OSCARSS has ceased , at which point all data entered into the database will be cleaned and locked. Only demographic and clinical data will be collected at study entry – data on any of our outcome measure scales cannot be considered to be ‘true baseline’ data as the intervention is implemented at a cluster level before the carer is recruited to the study. It is likely that the carer will already have received some components of the intervention by the time they consent to provide data. Study entry data will be accepted up until point receipt of data at the 3 month time point. This can occur if 3 month timepoints approach before ‘study entry’ data are collected - due to delays in receiving referrals or achieving consent.

### Table 2 outline of collected data to be included in cRCT statistical analysis collected

| **Measure** | **Method** | Carer Study entry (A) | date first intervention/ Control + **3 mnths ¥** | date first intervention/ Control + **6 mnths** |
| --- | --- | --- | --- | --- |
| Demographic & clinical data (as described section 2.2) | Postal / Telephone Carer self-report questionnaires | **X** |  |  |
| Caregiver Strain subscale of FACQ**†** |  |  | **X** | **X** |
| Caregiver distress subscale of FACQ |  |  | **X** | **X** |
| Positive caregiving appraisals subscale of FACQ |  |  | **X** | **X** |
| Pound Satisfaction with Stroke Services Scale |  |  | **X** | **X** |
| Hospital Anxiety and Depression Scale |  |  | **X** | **X** |

¥ 3 months = primary end point for carer quantitative outcomes

†Primary outcome

Acronyms used: FACQ = Family Appraisal of Caregiving Questionnaire (FACQ)

Data from service client management databases will also be sought at the end of the data collection period for all consented carers to provide information on the number, type and duration of support contacts received by each consented carer. Similar – and fully anonymised – datasets will also be sought that show whole service delivery at participating clusters. Cluster level datasets will be used to support exploration of the representativeness of contacts received by consented carers.

- 1. Missing data

**Primary outcome**

The Caregiver Strain subscale of the FACQ consists of 8 questions, each given a score out of 5. Data for any participant will be included in the primary outcome as long as a participant has given a single response to at least 6 of the 8 questions. Any missing values (either due to non response, multiple non-adjacent response or illegible response) will be replaced with the mean score on the remaining questions for that individual. Where multiple responses are adjacent i.e. carer has ticked Likert boxes 2 and 3, a random number generator is used to enter either the lower or higher of the two values at random. Any individual who has answered less than 6 questions at 3 months will be recorded as having missing data for the primary outcome. Reasons for missing data on the primary outcome will be recorded and reported in the CONSORT flow diagram – e.g. no follow up data provided, withdrawal, death, incomplete follow up data (less that 6 out of 8 questions on FACQ subscale). Analysis will be performed using all non-missing data.

**Secondary outcomes**

All analysis of secondary outcomes will be undertaken on the basis of ‘all available data’ per analysis.Where data are missing on 25% or fewer of values for any of the scales, any missing values will be imputed using the mean of the remaining values for that individual. Where data are missing for more than 25% of values for a particular scale, the participant will be recorded as having missing data for that outcome. Numbers missing for each outcome will be reported.

**Covariates**

Missing covariate data – will be imputed using simple imputation (White and Thompson 2005) using the remaining individual level covariates .

- 1. Assumption checking

Prior to unblinding the treatment allocation, we will check the distribution of the primary and secondary numeric variables to check whether they satisfy the assumptions for multilevel linear regression. Where appropriate, we will consider performing transformations. After analysis, assumptions will be confirmed using residual plots.

- 1. Data validation

The database manager will provide the statistician performing the analysis with an initial version of the full database without any group identification variable. The statistician will create a dummy variable for group, and the analysis will be prepared using this dataset. This will allow data validation and analysis issues to be discussed and resolved without knowledge of the group allocation.

1. Analysis of Outcomes
   1. Data manipulations

The Caregiver Strain subscale of the FACQ consists of 8 questions, each given a score out of 5. These 8 scores will be added together to come up with a total score, using the rules for missing data described in 3.7. Similar manipulation will be performed for the relevant secondary outcomes.

- 1. Data Analysis

Analysis of the primary outcome comparing intervention and control at three months would be performed using a multilevel regression model, with a random intercept for ‘site’ to take into account clustering and a fixed covariate for ‘intervention’ along with adjustment using the following fixed individual level covariates: stroke severity of cared-for’s stroke, time post-stroke, age of carer, health of carer (pre-existing long-term health conditions) and the following cluster level covariates: size of service, pre-existing knowledge/experience of staff delivering support. By the design of this cluster randomised trial, recruitment of individual carers takes place post randomisation and therefore we expect a certain amount of selection bias. We plan to adjust for baseline covariates in an attempt to control for any baseline imbalance.

Similar analysis will be used for all numeric secondary outcome measures.

The mean number of carers per cluster, the mean number of support contacts per carer per cluster and the mean duration of contacts per carer per cluster will be compared between control and intervention groups using t-tests. We would not expect these variables to have appropriate distributions for analysis using a linear mixed model.

- 1. Sensitivity analyses

We will undertake sensitivity analysis to assess any potential bias in the analysis of the primary outcome measure and examine how robust the findings are.

1. Without adjustment for covariates
2. Per protocol

Excluding individuals or whole clusters that did not adhere to the protocol as per section 3.4

1. Combining 3 and 6 month data

Using ‘time’ and ‘time by group interaction’ as fixed covariates, all available 3 month data and 6 month data will be combined for the Caregiver Strain subscale of the FACQ outcome This will allow us to explore how caregiver strain changes over time and whether any effect of the intervention changes over time.

1. Multiple imputation

Using multiple imputation to replace missing values on the primary outcome measure using the following covariates: stroke severity of cared-for’s stroke, time post-stroke, age of carer, health of carer (Royston 2005). This will be attempted for

(a) missing questions where a participant has provided a partial response

(b) missing mean score where a participant has provided no primary outcome data

1. Excluding delayed responses

Excluding any data from individuals who return their 3 months outcome data later than 4.5 months post date seen or 6 month outcome data later than 7.5 months post date seen.

1. Multiple carers of the same stroke survivor

Where multiple carers of the same stroke survivor have provided outcome data; excluding data from the second and subsequent carers linked to the same stroke survivor

- 1. Sub-group analysis

We are not planning any sub-group analysis over-and-above sensitivity analyses.

- 1. Safety data

It is not anticipated that any AE or SAE will be related to the intervention. The number of AE or SAE per arm as defined in the protocol will be reported.

- 1. Statistical Software

Analyses will be undertaken in Stata version 14(2) or later.

1. Health Economics components

The health economics evaluation will consider costs and health benefits (Quality Adjusted Life Years (QALYs)). Costs to deliver the intervention and costs associated with use of other healthcare and social care resources will be included. The outcome of the economic evaluation will be an Incremental cost-effectiveness ratio (ICER) which is a single figure which combines the costs and benefits of an intervention

- 1. Health Economics-specific data

Additional Health Economics- related data would be obtained in the 3 and 6 month follow-up questionnaires:

- Caregivers health utility given by EQ5D-5L
  - EQ5D-5L will also be collected at the study entry. As per the protocol description, study entry data can be collected at the same time as 3 month follow up questionnaires. In these cases, a merged postal pack only asks for EQ-5D-5L to be collected once and returned EQ-5D data are entered as 3 month data with ‘missing data’ recorded for study entry EQ-5D.
- Caregiver health service utilisation
  - The questionnaire from this study asks respondents whether they used the following health care services; GP, A&E / urgent care / walk-in centres, physiotherapist, psychiatrist, counsellor/psychologist and social worker. Respondents were also given the opportunity to record other health care services not listed.
- Caregiver estimates of informal care provision (amount and type of informal care provided).
- Financial support and benefits claimed for carers and the person they care for.

Additional data to be used in Health Economics analysis is provided by the Stroke Association at study end. These data log Stroke Association coordinator activity with each respondent; capturing information on duration, location and type of contact provided. In addition, a fully anonymised dataset showing participating Stroke Association coordinator activity with all clients in randomised services will be used in secondary analysis (see 5.4)

- 1. Missing data

Complete case analysis will be conducted with the assumption that the missing data fields are missing completely at random. Sensitivity analysis around the missing data for costs and QALYs issue are shown in section 5.3.4.

To ensure replicability of the work, we will document any occurrences of multiple and seemingly erroneous data from each respondent. At least two health economists will consult to agree consensus about decision rules to deal with these data.

- 1. Analysis of outcomes
     1. Unit Costs

Unit costs for resource used will be obtained through the Personal Social Services Research Unit (PSSRU), unit costs of health and social care (Curtis, 2017) and the NHS reference cost database.

A log for each respondent containing the duration of support provided by Stroke Association will be provided. This will be combined with the unit costs of Stroke Association coordinators time (includes all on costs associated with service delivery) to estimate the cost of delivering the intervention. Unit costs per hour of Stroke Association staff will be the same for both control group and intervention group staff. Time spent in staff training to deliver the intervention will be added to the intervention group only.

- - 1. Data Manipulations

EQ5D-5L responses will be converted into an EQ5D utility score, using the methodology that is in line with NICE guidance at the time of the analysis.

- - 1. **Data analysis**

Regression analysis will be used to estimate differences in costs and QALYs between the treatment and control groups. As clusters were randomised between treatment and control groups and not the respondents, this analysis will control for study entry differences in age, study entry EQ5D health, stroke survivor severity and any key differences in carers between the two arms of the intervention. We will also cluster on Stroke service site. We will use the same regression specification when estimating QALY and costs.

- - - 1. QALYs

EQ5D health utility scores at study entry, 3-months and 6-months will be used to generate Quality Adjusted Life Year (QALY) for each respondent using Area Under the Curve (AUC) (Hunter et al, 2015). AUC estimate of the QALY for each respondent is the average EQ5D utility value over the trial period. We will conduct regression analysis to estimate the average difference in QALY (net QALY) between treatment and control group.

- - - 1. Costs

Costs from health and social service utilisation and costs from Stroke Association coordinators time will be summed to generate a total cost for each respondent. Training costs of intervention will be added to respondents within the treatment arm of the intervention. Regression analysis will estimate the average difference in cost (net cost) between respondents in the intervention and control arm of the study.

- - - 1. ICER

The Incremental Cost-Effectiveness Ratio will be calculated using the net cost (5.3.3.2) divided by the net benefit (5.3.3.1). The ICER will be the cost of an additional QALY gained from the treatment, which will be used to determine whether the intervention is cost effective.

Respondent level costs and EQ5D health utility will be bootstrapped with replacement, N=10,000, which will be plotted on a cost effectiveness plane to visualise uncertainty. The probability of whether the intervention is cost effective at different Willingness To Pay Thresholds (WTPT) will be visualized using a cost-effectiveness acceptability curve (CEAC) (Briggs, 1997). A WTPT is the maximum amount of money to pay per QALY before interventions or technologies are deemed not cost effective as the same money could be spent elsewhere to generate a larger number of QALYs.

- - 1. **Sensitivity analysis of Economic Evaluation**

Multiple imputation using chained equations will be used to replace missing values for costs and QALYs in a sensitivity analysis (Leurent et al, 2018). A separate sensitivity analysis will be to include a per protocol analysis where we remove respondents that are removed from the ‘per-protocol’ sensitivity analysis (section 4.3). A further sensitivity analysis will be conducted which will remove respondents that are calculated as outliers in terms of high service users that may unduly skew the data.

A final sensitivity analysis will use the primary outcome measure of the study as mentioned in section 1.1 as the measure of health benefit. An ICER estimating the cost per unit increase on the Caregiver Strain subscale of the FACQ will be reported.

- 1. Secondary analyses
     1. **Valuing informal care**

A secondary analysis will value the informal care provision reported by respondents and explore whether the intervention has affected informal care provision. Value of informal care provision between intervention and control will be compared but this will not inform the primary economic evaluation.

Informal care will be valued using the proxy good method. The proxy good method substitutes the tasks performed by informal carers with unit costs of health care professionals using the PSSRU. Informal care is captured in four broad categories of tasks: “household”, “personal”, “health care” and “other”. The hours associated with the “household” tasks will be given the salary of a low-grade professional salary whereas “personal” and “health care” tasks will be grouped and attached to a higher skilled professional salary. For this analysis, hours captured in the “other” tasks will not be used in the valuation of informal care, but, it will be used as completeness check for the three categories. Additional sensitivity analysis will adjust for major outliers in this dataset (e.g. removing those who report providing more than 18 hours of informal care during a day)

- - 1. **2 year Modelling**

Due to the short 6-month follow-up period from the data collection, we propose a secondary analysis to extrapolate the data for up to two years beyond the data collection period, if the data are adequate. A discount rate will be used on values which have been extrapolated using a discount rate of 3.5% per annum which is in line with the NICE reference case.

- - 1. **Service provider cost comparison**

We will use the fully anonymised Stroke Association dataset of coordinator activity with all clients in the service (not just consent carer respondents). This will explore generalisability / representativeness of the sample of consented carers (in terms of coordinator time spent), but not inform the economic evaluation.

1. References

Briggs AH, Wonderling DE, Mooney CZ (1997). Pulling cost-effectiveness analysis up by its bootstraps: a non-parametric approach to confidence interval estimation. Health Economics (6). 327-340

Campbell MK, Piaggio G, et al. (2012). Consort 2010 statement: extension to cluster randomised trials. BMJ 345: e5661.

Cooper BGJ, Kinsella et al. (2006). Development and initial validation of a family appraisal of caregiving questionnaire for palliative care. Psychooncology 15(7): 613-622.

Curtis LA, Burns A. (2017) Unit Costs of Health and Social Care 2017. Report number: <https://doi.org/10.22024/UniKent/01.02/65559>. Personal Social Services Research Unit, University of Kent, 260 pp. ISBN 9781911353041.

Ewing G, Grande G, et al. (2013). Development of a Carer Support Needs Assessment Tool (CSNAT) for end-of-life care practice at home: a qualitative study. Palliat Med **27**(3): 244-256.

Farrin A, Russell I, et al (2005). Differential recruitment in a cluster randomized trial in primary care: the experience of the UK back pain, exercise, active management and manipulation (UK BEAM) feasibility study. Clin Trials. 2(2):119–124

Forster A, et al (2013). A structured training programme for caregivers of inpatients after stroke (TRACS): a cluster randomised controlled trial and cost-effectiveness analysis. Lancet. **382**(9910): p. 2069-76.

Giraudeau B, Ravaud P (2009). Preventing Bias in Cluster Randomised Trials. PLOS Med 6(5): e1000065

Hunter RM, Baio G, Butt T, Morris S, Round J, Freemantle N (2015). An Educational Review of the Statistical Issues in Analysing Utility Data for Cost-Utility Analysis. PharmacoEconomics. 33, 355-366

Leurent B, Gomes M, et al (2018). Sensitivity Analysis for Not-at-Random Missing Data in Trial-Based Cost-Effectiveness Analysis: A Tutorial. PharmacoEconomics (36). 889-901

Leyrat C, Caille A, Donner A, Giraudeau B (2013). Propensity scores used for analysis of cluster randomized trials with selection bias: a simulation study. Stat Med. 32(19):3357–3372

Royston P (2005). Multiple imputation of missing values: update of ice. Stata Journal. 5 (4) 527-536.

Seaman S, White I (2011). Review of inverse probability weighting for dealing with missing data. Statistical Methods in Medical Research. 22 (3) 278-295.

White IR, Thompson SG. (2005) Adjusting for partially missing baseline measurements in randomized trials. Stat Med. 24 993–1007.
